# Data citation: what, when, why?

**DOI:** 10.15252/msb.20188783

**Published:** 2018-12-17

**Authors:** Thomas Lemberger

**Affiliations:** ^1^ EMBO Heidelberg Germany

## Abstract

*EMBO Press* has implemented a structured Data Availability Section as well as formal data citations in research articles at all its journals. This serves to improve access to research data and provide a mechanism for credit attribution to data producers.

Biology has become a data‐intensive science with a broad variety of data types being produced, exchanged, and used across laboratories and databases. The open sharing of research data among collaborators and with the research community at large creates many opportunities for researchers to learn details about specific results, to perform additional analyses, and to generate hypotheses and make discoveries by integrating multiple datasets. A key element in this emerging Open Science research environment is a general mechanism that permits referencing of stably archived datasets in a way that provides users with direct access to the data while attributing credit to the data producers. Integration of datasets with research papers will play a crucial role in assuring the reproducibility of the research papers.

To implement a scholarly referencing and credit system for data, *EMBO Press* has introduced in all its journals, including *Molecular Systems Biology*, two new Open Science policies:

- A structured Data Availability Section.
- A formal data citation format in the article reference list.

This initial implementation of data citation at *EMBO Press* is based on the work of the Early Adopter Expert Group, a group of publishers who recently delineated an early implementation roadmap for data citations (Cousijn *et al*, 2018).

## The data availability section

All too often, accession numbers to primary datasets are buried in Material & Methods, supplementary information, or other parts of the paper. The purpose of the Data Availability Section is therefore to provide direct access to the novel primary data which were produced in the context of the study and are essential to support its conclusions. Each dataset will be listed with the following elements: (i) the repository where the data have been deposited (see also our data deposition policies: http://msb.embopress.org/authorguide#datadeposition); (ii) the unique identification number (usually an accession number or a DOI) of the dataset within the database; and (iii) a resolvable link that directly leads to the dataset. Importantly, this link should point to the landing page where the dataset can be directly accessed, as opposed to a generic link leading to the home page of the database (see guidelines at http://msb.embopress.org/authorguide#dataavailability). The Data Availability Section is mandatory for all papers that report new datasets for which deposition is requested (see http://msb.embopress.org/authorguidelines/datadeposition).

## Data citations

In studies that reuse previously released public data for comparison, re‐analysis or data integration, we encourage authors to include formal data citations in the reference list in addition to classical bibliographical references. Data citations will directly link to the database record where the respective dataset can be accessed.

Data citations are distinct from, and complementary to, classical bibliographical citations. Citing the relevant research papers remains a fundamental rule of good scholarly practice. Indeed, a scientific paper is not only limited to the reporting of primary data. Documenting the methods used to obtain the data and providing the context, motivation, and reasoning for the generation and the interpretation of the data are essential components of a fully fledged scientific study. As such, we envision data citations as a useful additional mechanism that will co‐exist with literature citations. We regard it as best practice to cite both the data and the associated research paper where appropriate.

The format of data citations is similar to bibliographical references: Instead of a journal, the reference includes the name of the database; instead of volume and page number, it provides an accession number. If a dataset does not have a title or is anonymous, it can nevertheless be included for reference. Importantly, since a dataset is usually not peer‐reviewed and is a fundamentally different type of research object than a peer‐reviewed article, data references should be clearly labeled with the “[DATASET]” keyword at the end of the reference. Detailed information and examples are provided in our authors guidelines (http://msb.embopress.org/authorguide#datadeposition). Under the hood, data citations are encoded in a specific way to make them machine readable (Mietchen *et al*, 2015) and are collected by CrossRef.

*A priori*, links to novel primary data and to previously published data could have all been aggregated into the reference list. Consultation with our community, however, indicated a overwhelming preference for a distinct Data Availability Section dedicated to primary data and for separate data citations in the reference list to link to previously published or deposited data.

## What's next?

*EMBO Press* adopted the i4OC open reference format to make reference lists openly accessible (https://i4oc.org) and we have no size limitation for the reference list. Implementing data citations is extending further our efforts in improving the utility of citations. It is also part of a broader multi‐pronged effort at *EMBO Press* to improve access, reproducibility, and utility of research data in scientific papers. This includes the recent introduction of Structured Methods (Polychronidou, 2018), the implementation of data quality and integrity checks (Pulverer, 2015), and the development of the SourceData project that makes the data behind figures searchable and accessible (http://sourcedata.io, Liechti *et al*, 2017). In a subsequent step, accession numbers to source data associated with figures will automatically be included in the Data Availability Section, thanks to the integration of SourceData with the BioStudies database (http://bit.ly/2G0Cn8R). This integrated system will implement FAIR data principles (Wilkinson *et al*, 2016) in publishing by inter‐linking figures based on their data content, making them findable and enabling direct download of the associated research data. These developments will be announced in further editorials across the *EMBO Press* titles.
